# A novel necroptosis pathway: orchestrated by non-canonical STING-ZBP1 signaling

**DOI:** 10.1038/s41392-026-02587-7

**Published:** 2026-02-18

**Authors:** Xiangyu Zhu, Sijia Liu, Long Zhang

**Affiliations:** 1 International Biomed-X Research Center, Second Affiliated Hospital of Zhejiang University School of Medicine, Zhejiang University, Hangzhou, China; 2 MOE Basic Research and Innovation Center for the Targeted Therapeutics of Solid Tumors, Institute of Biomedical Innovation, School of Basic Medical Sciences, Jiangxi Medical College, Nanchang University, Nanchang, China; 3 Key Laboratory of Precision Diagnosis and Treatment for Hepatobiliary and Pancreatic Tumor of Zhejiang Province, Hangzhou, China; 4 MOE Laboratory of Biosystems Homeostasis and Protection and Innovation Center for Cell Signaling Network, Life Sciences Institute, Zhejiang University, Hangzhou, China

**Keywords:** Cell biology, Molecular medicine

In a recent publication in Nature, Kelepouras et al. identified a novel necroptosis pathway in which activation of stimulator of interferon genes (STING) triggers Z-DNA binding protein 1 (ZBP1)-mediated cell death, independent of canonical tumor necrosis factor receptor 1 (TNFR1) and Fas-associated death domain protein (FADD) signaling (Fig. 1). These findings provide a transformative therapeutic paradigm by positioning the STING-ZBP1 necroptosis axis as a pharmacologically actionable target, offering a mechanism-based strategy to treat STING-associated vasculopathy with onset in infancy (SAVI) and related interferonopathies.^1^

**Fig. 1:**
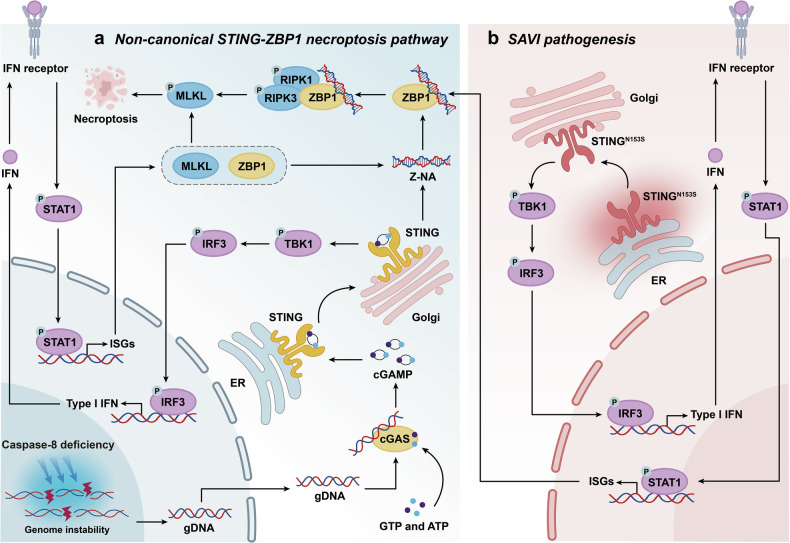
Schematic diagram of how non-canonical STING-ZBP1 signaling orchestrates the necroptosis pathway and SAVI pathogenesis. **a** The absence of caspase-8 leads to genomic instability and the production of gDNA. cGAS is activated by cytosolic gDNA and catalyzes cGAMP production. Upon binding to the ligand-binding domain of STING, cGAMP induces a conformational change in STING at the endoplasmic reticulum (ER). STING is then transported to the Golgi apparatus, where it phosphorylates TBK1. TBK1 activates IRF3, which induces the production of interferons (IFNs). Through the IFN receptor-mediated downstream signaling pathway, this ultimately leads to the phosphorylation and activation of STAT1. STAT1 subsequently upregulates the expression of ISGs (ZBP1 and MLKL). Concurrently, STING stabilizes the structure of intracellular Z-NA, promoting its accumulation. Upon recognizing Z-NA, ZBP1 undergoes a conformational change, recruiting and phosphorylating RIPK1 and RIPK3. pRIPK3 then activates the necroptosis effector protein MLKL, ultimately leading to necroptosis. **b** In STING-associated infantile vascular disease SAVI, *STING*^*N153S*^ becomes spontaneously activated independently of upstream cGAMP binding. Its protein localization shifts from the resting ER to Golgi, further recruiting downstream immune-inflammatory signals and activating the ZBP1-RIPK3-MLKL necroptosis pathway. This leads to abnormal expression of immune and inflammatory factors in SAVI. Genetic ablation of RIPK3 in the *Sting*^*N153S*^ SAVI preclinical mouse model could rescue the pathology, providing direct genetic evidence that STING-mediated necroptosis constitutes the core pathogenic mechanism of SAVI

Necroptosis is a highly programmed defense system against exogenous infection and self-inflammation. Nevertheless, its dysregulation acts as a double-edged sword, where excessive activation of necroptosis leads to severe cell death and inflammation. Cysteinyl aspartate-specific proteinase-8 (caspase-8) inhibits the key necroptosis proteins receptor-interacting protein kinase 3 (RIPK3) and mixed lineage kinase domain-like protein (MLKL), preventing excessive necroptosis activation and maintaining homeostasis.^2^ Conventional wisdom holds that necroptosis relies primarily on a TNFR1-mediated signaling pathway, which activates downstream MLKL for cell death via the FADD-RIPK1-RIPK3 complex. However, the mechanism of TNFR1-independent necroptosis in diseases caused by abnormal innate immune activation has not been fully investigated.

To further investigate the necroptosis signaling pathway, researchers have employed a caspase-8 epidermal knockout (*Casp8*^*E-KO*^) mouse model that develops lethal necroptosis-driven dermatitis. In line with previous findings, genetic knockout of either RIPK3 or MLKL completely rescued the lethal phenotype. Strikingly, deficiency in TNFR1 or FADD afforded only partial protection, suggesting the existence of a non-canonical necroptosis pathway. Through genetic and biochemical exploration, they discovered that caspase-8 deficiency disrupts genomic stability, leading to mitotic abnormalities, DNA damage, and leakage of genomic DNA (gDNA) into the cytoplasm. This cytoplasmic gDNA specifically activates cyclic GMP-AMP synthase (cGAS), a universal sensor for both exogenous and endogenous nucleic acids.^3^ Activated cGAS initiates the STING pathway, driving the sequential phosphorylation of TANK-binding kinase 1 (TBK1) and signal transducer and activator of transcription 1 (STAT1). This signaling cascade orchestrates the interferon (IFN) response transcription program, leading to the upregulation of interferon-stimulated genes (ISGs), including the key necroptosis effector molecules ZBP1 and MLKL.

Notably, the regulatory role of STING is not limited to promoting ZBP1 transcription. Immunofluorescence assays further demonstrated that STING activation also enhances the accumulation and stabilization of Z-form nucleic acid (Z-NA). These dual functions of STING synergistically establish a signaling environment that ensures robust ZBP1 activation. Previous studies have shown that Z-NA sensing activates ZBP1 and recruits RIPK3 to trigger necroptosis, but this process is normally suppressed by RIPK1 through the FADD-mediated recruitment of caspase-8, which cleaves RIPK1 and RIPK3 to inhibit cell death.^4^ This study defines a non‑canonical necroptosis pathway induced by STING activation under conditions of caspase‑8 deficiency. This pathway operates via a ZBP1–RIPK1–RIPK3 complex and functions independently of the canonical TNF–TNFR1-induced FADD–RIPK1–RIPK3 signaling axis. The core regulatory role of STING in this pathway has been pharmacologically confirmed: the STING antagonist C-178 significantly suppressed both ZBP1 upregulation and RIPK1/3 phosphorylation, whereas the agonist DMXAA potently enhanced these events. In *Casp8*^*E-KO*^ mice, specific knockout of keratinocyte STING or systemic knockout of ZBP1 significantly delayed the onset of lethal dermatitis, reduced skin inflammation and pMLKL accumulation, and prolonged mouse survival. Unlike TNFR1 knockout, which only partially delays symptoms, STING or ZBP1 knockout markedly improves systemic inflammation (e.g., in the spleen and liver), suggesting that the STING-ZBP1 axis is a key driver independent of TNFR1 in the necroptosis pathway. These results revealed an important function of STING as an “upstream regulatory hub” for necroptosis, which provides a link between cytoplasmic DNA recognition and inflammatory cell necroptosis.

Gain-of-function mutations in STING cause a rare interferonopathy, SAVI, characterized by early-onset systemic inflammation, cutaneous lesions, and interstitial lung disease.^5^ Following conventional treatment, SAVI patients still exhibit elevated levels of the necrosis-associated cytokines IL-21, CXCL1, and IL-1β in their peripheral blood, alongside significant upregulation of necrosis-related genes, including ZBP1, MLKL, and RIPK3, which are coexpressed with type I interferon response genes. In the *Sting*^*N153S*^ SAVI preclinical mouse model, chronic STING activation persistently drives ZBP1-MLKL-dependent necroptosis. Knocking out RIPK3 significantly improved skin lesions, pulmonary inflammation, and immune cell abnormalities in mice, with markedly increased survival rates. These findings indicate that STING‑mediated necroptosis contributes to the pathogenesis of SAVI, suggesting that targeting the STING‑RIPK3 axis may contribute a therapeutic strategy for this disease. However, the authors did not perform MLKL knockout in the *Sting*^*N153S*^ SAVI preclinical mouse model. Thus, the possibility that SAVI can be mediated through MLKL-independent mechanisms cannot be excluded, and RIPK3 may also drive the pathological state of SAVI via alternative pathways.

In summary, this study reshapes our understanding of necroptosis signaling, confirming that STING serves as the pivotal node linking cytoplasmic DNA recognition to a non-canonical TNFR1/FADD-independent necroptosis pathway. By elucidating the cGAS-STING-ZBP1-RIPK3-MLKL axis, this study not only uncovers the mechanism of caspase-8-deficient inflammation but also proposes a novel interpretation for the pathophysiology of SAVI, suggesting a promising therapeutic concept for patients with severe inflammatory diseases such as SAVI. Given that STING gain-of-function mutations are also associated with other autoimmune diseases, this STING-ZBP1 necroptosis signaling pathway may elucidate the pathological mechanisms of additional disorders, paving new avenues for precision medicine for other refractory interferon-related diseases.

## References

[1] Kelepouras, K. et al. STING induces ZBP1-mediated necroptosis independently of TNFR1 and FADD. *Nature* **647**, 735–746 (2025). 40834903 10.1038/s41586-025-09536-4 PMC12629989

[2] Fritsch, M. et al. Caspase-8 is the molecular switch for apoptosis, necroptosis and pyroptosis. *Nature* **575**, 683–687 (2019). 31748744 10.1038/s41586-019-1770-6

[3] Sun, L., Wu, J., Du, F., Chen, X. & Chen, Z. J. Cyclic GMP-AMP synthase is a cytosolic DNA sensor that activates the type I interferon pathway. *Science* **339**, 786–791 (2013). 23258413 10.1126/science.1232458 PMC3863629

[4] Jiao, H. et al. Z-nucleic-acid sensing triggers ZBP1-dependent necroptosis and inflammation. *Nature* **580**, 391–395 (2020). 32296175 10.1038/s41586-020-2129-8 PMC7279955

[5] Liu, Y. et al. Activated STING in a vascular and pulmonary syndrome. *N. Engl. J. Med.* **371**, 507–518 (2014). 25029335 10.1056/NEJMoa1312625 PMC4174543

